# Slip length and structure of liquid water flowing past atomistic smooth charged walls

**DOI:** 10.1038/s41598-019-55491-2

**Published:** 2019-12-12

**Authors:** Xinran Geng, Miao Yu, Wei Zhang, Qiwei Liu, Xiaopeng Yu, Yang Lu

**Affiliations:** 1 Jilin Provincial Key Laboratory for Numerical Simulation, Jilin Normal University, Siping, Jilin, 136000 P. R. China; 2 School of Materials Science and Engineering, Institute for Advanced Materials, Jiangsu University, Zhenjiang, 212013 P. R. China; 3 Changchun No.6 Middle School, Changchun, Jilin, 130031 P. R. China

**Keywords:** Chemical physics, Fluid dynamics

## Abstract

In this work, the slip behavior and structure of liquid water flowing between two charged solid planar walls were investigated using non-equilibrium molecular dynamics simulations. The upper and lower walls are positive and negative charged, respectively. It was shown that the slip length increases at smaller water-solid interaction energy and become smaller with increasing the surface charge density. At the largest surface charge density, the slip length nearly independent of the water-solid interaction energy. The relationship between the slip length and surface charge density and water-solid interaction energy was rationalized by considering the static structure factor of liquid water. Interestingly, the positive charged surface induces less ordering structure and larger slip at the small surface charge density than that by the negative charged surface. While, at large surface charge density, the opposite correlation is observed. Furthermore, we find that the relationship between the slip length and the normalized main peak of static structure factor collapses onto a single curve for different water-solid interaction energies and surface charge densities. The results of the present work open perspectives for modeling complex systems with combined effects of surface charge and wettability.

## Introduction

Since the end of last century, micro- and nanofluidic devices have greatly enhanced our ability to manipulate small volumes of fluid^1^. This has led to many applications for chemical analysis, biological characterization, cell capture and *et al*.^2^. The distinguished characteristic of micro- and nanofluidic devices is the large surface-to-volume ratio leading the boundary slip effect imposing significant influences on the flow properties^3^. The boundary slip was first analyzed by Navier in 1823. He proposed the so-called Navier slip model to quantify the boundary slip phenomenon by introduce the concept of slip length which defined as a distance from the boundary where the linearly extrapolated fluid velocity profile vanishes.

Many experiments^2,4–7^ and simulations^3,8–12^ demonstrated that the typical magnitude of the slip length is in the order of tens nanometers. Such small value of the slip length can be safely ignored in the macroflows, which leads to the no-slip boundary condition was widely used in the past centuries in the numerical simulation and theoretical analysis. However, the magnitude of slip length has significant influence on the flow properties in micro and nano scale^13,14^. The experimental studies of slip length are difficult because it is very hard to resolve the fluid velocity profile in the region near the liquid/solid interface at these length scales^15^. Alternatively, molecular dynamics (MD) simulations have widely used to investigate the slip properties of liquid flowing past solid surface since it can resolve the velocity profile from the atomistic level^13,14,16–27^. Moreover, there are no assumptions about the slip velocity at the interface are required.

It has been found that the degree of slip at liquid-wall interface is mainly controlled by the liquid-wall interaction, the degree of commensurability of liquid and solid structures at the interface, and diffusion of fluid molecules near the wall. It is intuitive to understand that the slip length inversely depends on the liquid-wall interaction, which was demonstrated by many MD studies^3,10,12,16,17,19,28,29^. However, Hu *et al*.^30^ show that this is not a universal case, that is, the slip length is positively correlated to the liquid-wall interaction when the liquid-wall interaction is small enough. A significant advance understanding of the slip is that the slip length is well correlated with the main peak of static structure factor in the first fluid layer^3,16,17,19^. Furthermore, it was found that the slip length is proportional to the collective relaxation coefficient of the fluid molecules near the wall for weak liquid-wall interactions and smooth surfaces^19^.

Besides the general understanding slip of simple liquid over solid surface, the liquid water over solid substrate attracted abiding interesting because of the practical importance^8,21,22,27,28,31,32^. Sendner *et al*.^22^ examined the slip behavior and structure of water at hydrophobic and hydrophilic diamond surfaces via nonequilibrium MD simulations. They found that the slip length negatively depends on the water-solid interaction strength. Moreover, they proposed a heuristic scaling relations between slip length, contact angle, and depletion layer thickness. Falk *et al*.^31^ studied the slip behaviors of water at graphitic interfaces with various topologies to disentangle confinement and curvature effects on slip. A strong curvature dependence of slip length was identified. At the same water-solid interaction energy, the carbon nanotube produces larger slip compared with the graphene slab. They further analyzed this curvature dependence by considering the water ordering structure that shows a curvature-induced incommensurability between the water and carbon structures.

The distinguished characteristic of slip of liquid water over solid surface is that the water molecule is highly polar. Therefore, liquid water will exhibit unique properties when it is in contact with a charged surface. Also, the charged surface produces an addition approach to control the water flow properties at micro- and nanoscale. Yoshida *et al*.^29^ examined the electrokinetic flows of an aqueous NaCl solution in nanochannels with negatively charged surfaces using MD simulations. They found that the larger surface charge density decreases the transport coefficients because the charged surface can strongly bound the counter-ions onto the vicinity of solid surface. Celebi *et al*.^33,34^ investigated the influence of surface charge density on the deionized water flow through positively charged graphene nano-channels using MD simulations. They found that the slip length decreases with the increasing surface charge density. In addition, they notice that the water molecules reorient their dipoles with oxygen atoms facing the positively charged surfaces. The previous studies^3,16,22^ show that the water-solid interaction energy is also significantly influence the liquid density and slip length of liquid water over solid surface. However, the combination effects of surface charge density and water-solid interaction on the water structure and slip properties are not systematically investigated.

In this work, we investigate the slip behaviors and structure of liquid water flowing between two charged solid planar walls using non-equilibrium MD simulations. Both the positive and negative charged walls are considered. The slip behaviors and structures of liquid water near walls are detailed investigated under different water-solid interaction energies and surface charge densities. The slip length increases at smaller water-solid interaction energies and surface charge densities. At the largest surface charge density, the slip length nearly independent of the water-solid interaction energy. Interestingly, we find that the positive charged surface induces larger slip at the small surface charge density than that by the negative charged surface. While, at large surface charge density, the opposite correlation is observed. The structure of liquid water was studied by considering the static structure factor of within in the first liquid water layer, which is used to rationalize the slip behaviors. The surface charge increases the ordering structure of liquid water leading to the decrease of slip length. The increase of the surface charge density changes the dependence of the main peak of the static structure on the water-solid interaction energy from nonlinear relationship to linear relationship. This is the origin of the dependence of slip length on the water-solid interaction energy. Furthermore, we identified a universal relationship between the main peak of static structure factor at different water-solid interaction energies and surface charge densities. The results of the present work open perspectives for modeling complex systems with combined effects of surface charge and wettability.

## Methods

**Figure 1:**
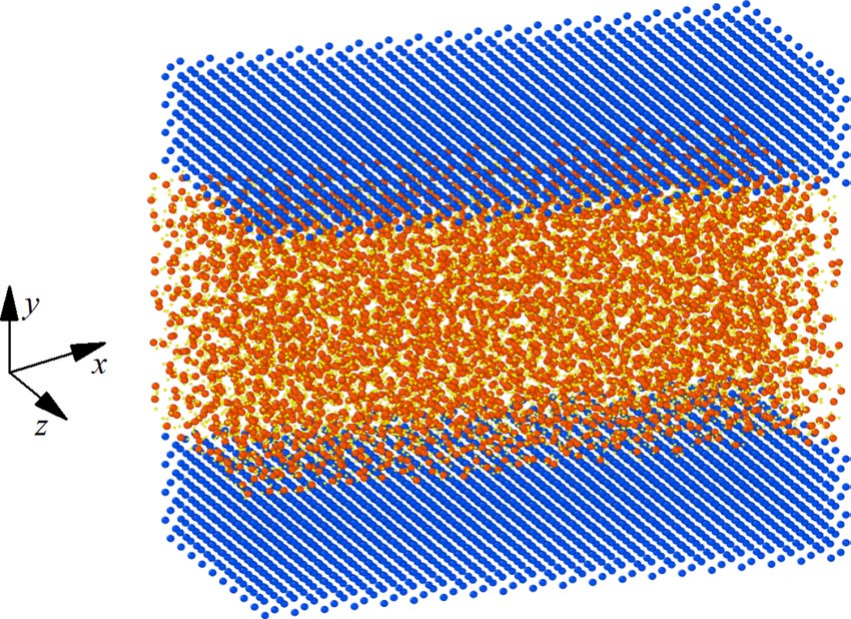
The simulation model of liquid water confined between two planar atomistic smooth wall. Each wall consists of 3200 atoms. There are 4394 molecules in liquid water.

We consider Poiseuille liquid water flows confined between two charged planar solid walls as shown in Fig. 1. Each wall is made of four layers constructed as face-centered-cubic structure with lattice constant of 4.05 Å. Solid walls were oriented on the *xz* plane. Dimensions of the simulation domain were set as 7.98 × 6.84 × 3.85 nm in the lateral (*x*) and vertical (*y*) and longitudinal (*z*) directions, respectively. The channel height is *h* = 4.0 nm, which is large enough to produce a bulk region in the center of channel^27,33,34^.

The interaction between atoms were modeled using Lennard-Jones (LJ) and Coulomb potentials given by,

1 $${\rm{U}}({r}_{ij})=4\varepsilon \,[{(\frac{\sigma }{{r}_{ij}})}^{12}-{(\frac{\sigma }{{r}_{ij}})}^{6}]+\frac{1}{4\pi {\varepsilon }_{0}}{\sum }_{i}^{a}\,{\sum }_{j}^{b}\,\frac{{q}_{i}{q}_{j}}{{r}_{ij}}$$

where *ε* and *σ* are the characteristic energy and length of the LJ potential. *ε*_0_ is the vacuum permittivity, *q*_*i*_ is the particle charges, and *r*_*ij*_ is the distance between two atoms *i* and *j*. The interaction potential parameters between Oxygen atoms are *ε*_*oo*_ = 0.1553 Kcal/mol and *σ*_*oo*_ = 3.166 Å. The LJ interactions involve Hydrogen atoms are set as zero. The mass of Oxygen and Hydrogen atoms are 15.9994 g/mol and 1.008 g/mol. The charge of Oxygen and Hydrogen atoms are **−**0.820*e* and 0.410*e*, where *e* is the charge of a proton. The LJ characteristic length between Oxygen and solid atoms is *σ*_*oo*_ = 3.166 Å. The interaction energy between Oxygen and solid wall atoms (*ε*_*wo*_) is changed to study the influence of wettability of solid surface on the flow properties.

Water molecules were modeled using a rigid SPC/E model. We used SHAKE algorithm^35^ to keep the bond length and angle of water molecules rigid. There is no interaction between solid atoms to model a rigid wall model, which can be used to improve the computational efficiency. The charge was equally distributed on the solid atoms at the innermost layers, and the rest solid atoms are neutral. The upper wall is positive charged and the lower wall is negative charged, which makes the system to satisfy the neutrality of the simulation box, thus, the system can be accurately solved by the Particle-Particle-Particle-Mesh (PPPM) algorithm^36^. Also, the influence of both the positive and negative charge on the slip behavior and structure of water can be considered in a single system. The surface charge density (CD) consider in the work is *CD* = 0 *μ*C/cm^2^ to 26.24 *μ*C/cm^2^, which is the similar to the previous study^33,34,37,38^. We used a cutoff distance of 1 nm for all LJ calculations and Coulomb potential. The Coulomb potentials is solved using the Particle-Particle-Particle-Mesh (PPPM) algorithm^36^.

The planar Poiseuille flow was induced by applying a constant acceleration force *a*_*x*_ to each Oxygen and Hydrogen atom in the +*x* direction. At the beginning of the simulation, the Nosé-Hoover thermostat was applied to the water molecular at the temperature of 300 K and the bulk density of water at 1000 kg/m^3^. After additional 10^6^ MD time steps, the constant acceleration force was applied to Oxygen and Hydrogen atoms. The thermostat was only applied in the direction perpendicular to the flow direction. The time interval of 10^6^ MD time steps was used to reach the steady Poiseuille flow. The velocity and density profiles were averaged within slices of thickness Δ*y* = 0.2 Å for additional 2 × 10^6^ MD time steps. All MD simulations were carried out using the open-source LAMMPS MD code^39^ with the time step Δ*t* = 1 fs.

The slip length was computed using the Navier slip model, ${L}_{S}\,={v}_{{\rm{S}}}/\dot{\gamma }$, where *v*_S_ and $\dot{\gamma }$ are the slip velocity and shear rate at the water-solid interface. The locations of the interfaces were defined at the lattice positions of the bottom layer of the upper wall and the top layer of the lower wall. To obtain the parameters, *v*_S_ and $\dot{\gamma }$, we firstly fit the velocity profile in the central part of the channel using a parabolic function. Thus, we will get the mathematical expression of the velocity profiles, as well as expression of the shear rate profile. Finally, we take the position of the water-solid interface into the corresponding expression to obtain the value of *v*_S_ and $\dot{\gamma }$, respectively. This procedure used to calculate the slip length is the same as previous studies^3,13–19,21,25,27^.

## Results and Discussions

### Density and velocity profiles

**Figure 2:**
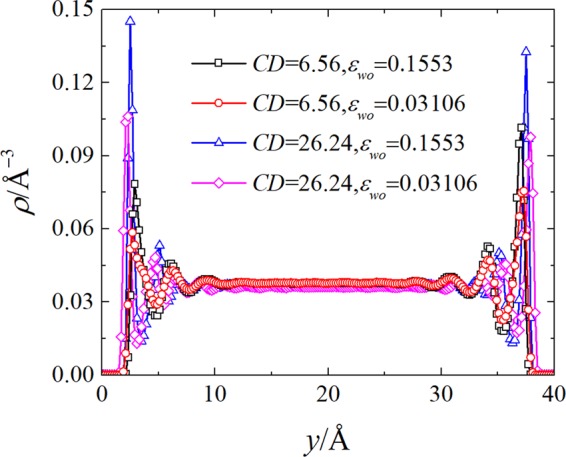
The density profiles for the indicated surface charge densities (*CD*) and water-solid interaction energies (*ε*_*wo*_).

Figure 2 presents the representative density profiles under different surface charge densities and water-solid interaction energies. It can be seen from the Fig. 2 that the density profiles exhibit profound decaying oscillation near two walls, and in the center part of channels, the density of water is equal to the expected values. The first peak of density profiles adjoining the walls is the so-called contact density, which is closely related to the magnitude of slip length^16,23^. For given surface charge density, increasing the water-solid interaction produces larger contact density. And, larger surface charge density also increases the magnitude of contact density for give water-solid interaction energy. In addition, the presence of surface charge changes the oscillation phase of density profiles. It is interesting to note that the negative and positive surface charge has different influence on the density profile. At small surface charge density (*CD* = 6.56 *μ*C/cm^2^), the positive surface charge induces larger contact density than that in the case of negative charged surface, while, at large surface charge density (*CD* = 26.24 *μ*C/cm^2^), the opposite trend is observed. A correlation between the liquid water structure near the wall and the slip length will be examined in the next section.

For nanoconfined fluids, the velocity profiles in the central part of the channel are well described by the continuum fluid dynamics^3,16,30,40^. To remind, the solution of the Navier-Stokes for incompressible steady Poiseuille flow without slip BC are given by

2 $${v}_{x}(y)=\frac{\rho {F}_{x}{H}^{2}}{2\mu }[\frac{1}{4}-{(\frac{y}{H}-\frac{1}{2})}^{2}],$$

where *H* is the channel height. Here, *μ* are the fluid shear viscosity. However, it was shown that Eq. (2) are modified by the velocity slip at the water-solid interface when the surface charge density or water-solid interaction energy are varied^3,16,30,40^.

**Figure 3:**
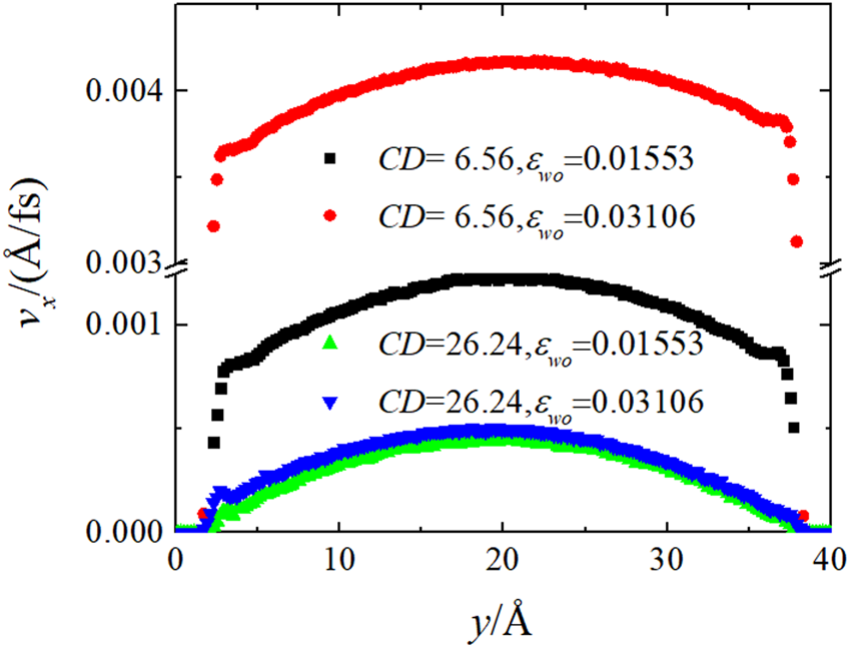
The velocity profiles for the indicated surface charge densities (*CD*) and water-solid interaction energies (*ε*_*wo*_).

Figure 3 shows representative velocity profiles in steady-state flow for selected values of surface charge density and water-solid interaction. As is evident, the liquid water velocity profiles in the center part of channel are well fitted by a parabola, as predicted by the continuum hydrodynamics [see Eq. (2)]. It can be clearly seen that the slip velocity *v*_S_ increases with the decreasing water-solid interaction energy for the surface charge density *CD* = 6.56 *μ*C/cm^2^. By sharp contrast, the slip velocity only slightly increases with the decreasing water-solid interaction energy for the surface charge density *CD* = 24.64 *μ*C/cm^2^. At given water-solid interaction, *ε*_*wo*_ = 0.1553 Kcal/mol and 0.03106 cal/mol, increasing the surface charge density decreases the slip velocity. It is also interesting to note that the positive charge produces larger slip velocity than that by the negative charge for the surface charge density *CD* = 6.56 *μ*C/cm^2^. While, the negative charge produces larger slip velocity than that by the positive charge for the surface charge density *CD* = 24.64 *μ*C/cm^2^. A more detailed analysis of the slip behavior for different surface charge density and water-solid interaction energies will be presented in the next section.

### Slip behaviors for different surface charge densities and water-solid interaction energies

**Figure 4:**
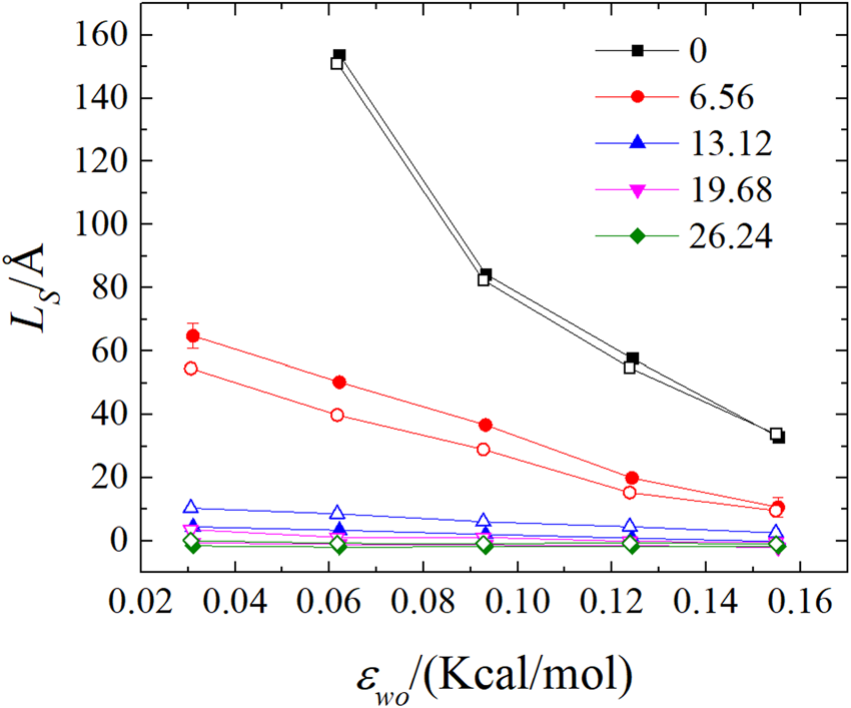
The slip length for the indicated surface charge densities (*CD*) and water-solid interaction energies (*ε*_*wo*_). The closed and open data point indicate the slip length calculated at the positive and negative charge walls, respectively.

The variation of the slip length with increasing water-solid interaction for different values of the surface charge density is presented in Fig. 4. The data for neutral solid walls, are also shown in Fig. 4 for comparison. For neutral solid walls, the slip length increases with the decreasing water-solid interaction energy. The relationship between the slip length and water-solid interaction is nonlinear, and the dependence of slip length can be fitted using a quadratic function, see the black squares in Fig. 4. As the surface is charged, the slip length is decreased for given water-solid interaction energy, which is consistent with the velocity profiles shown in Fig. 3. Increasing surface charge density weakens the influence of water-solid interaction energy on the slip length. As the surface charge density increases to $CD\gtrsim 19.68\,\mu {\rm{C}}/{{\rm{cm}}}^{2}$, the effect of water-solid interaction energy on the slip length becomes negligible. Moreover, the magnitude of the slip length is significantly reduced under large surface charge density. At the largest surface charge density, the boundary condition of water become no-slip. Notably, the presence of surface charge changes the nonlinear dependence of slip length on the water-solid interaction energy to linear manner. This linear function relationship between the slip length and water-solid interaction energy is well hold for the surface charge density, *CD* = 6.56 *μ*C/cm^2^ to 24.64 *μC*/cm^2^. Being consistent with the influence of surface charge on the velocity, the positive charge produces larger slip length than that by the negative charge for the surface charge density *CD* = 6.56 *μ*C/cm^2^. While, the negative charge produces larger slip length than that by the positive charge for the surface charge density *CD* > 6.56 *μ*C/cm^2^.

### Analysis of slip behaviors from the static structure factor

The slip behaviors of liquid water can be understood by examining the ordering structure of liquid water at the water-solid interface. The induced structure of liquid water by the solid walls can be quantitatively measured using the concept of the in-plane static structure factor, *S*(**k**). It can be seen in the density profiles that the liquid water near the wall exhibit several layers and the in-plane static structure factor typically calculated using the atom positions within each layer. Thus *S*(**k**) is a quantitative measure of the in-plane ordering for each layer of water near walls^16,17,19^. It has been demonstrated in the previous MD studies^17,19^ that the slip length is inversely correlated with the magnitude of the main peak of the in-plane static structure factor of the first water layer (FWL), *S*(**G**_1_), where **G**_1_ is the shortest reciprocal-lattice vector. The first water layer is defined as the water molecules within the region between the wall and first minimum in the density profile^16,17,19^. The in-plane static structure factor is given by^16,17^

$$S({\bf{k}})=\frac{1}{N}{|{\sum }_{j}{e}^{i{\bf{k}}\cdot {{\bf{r}}}_{j}}|}^{2},$$

where **r**_*j*_ = (*x*_*j*_, *z*_*j*_) is the two-dimensional position vector of the *j-*th atom and the sum is taken over *N* atoms within the FWL. Here, **k** = (*k*_*x*_, *k*_*z*_) is the reciprocal vector parallel to the walls. In a finite system, the components of the vector **k** are restricted to integer multiples of 2π/L, where *L* is the system size in the *x* and *z* directions. Thus, the larger the system size, the smaller the values of *k*_*x*_ and *k*_*z*_ can be.

**Figure 5:**
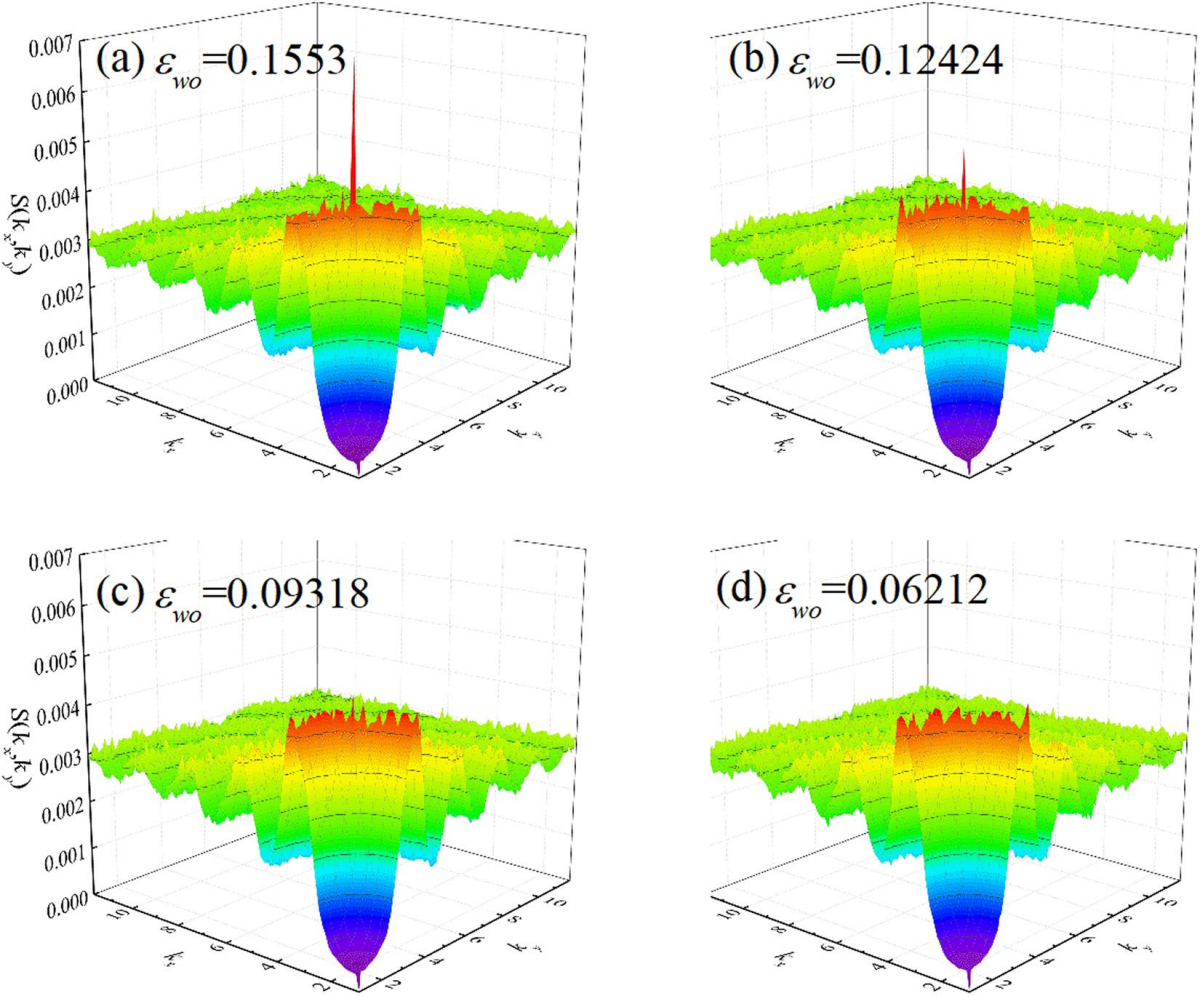
Examples of static structure factor with zero surface charge density for different water-solid interaction energy.

The quantity *S*(**G**_1_) depends on the system size and the number of atoms. In our simulations, the average number of fluid atoms in the FWL depends of the water-solid interaction energy. Therefore, the size-independent quantity *S*(**G**_1_)/*S*(0), averaged over 1 ns, was used to correlate the water structure with the slip length. We first shown examples of static structure factor with zero surface charge density for different water-solid interaction energy, as shown in Fig. 5. At strong water-solid interaction energy, *ε*_*wo*_ = 0.1553 Kcal/mol, the in-plane static structure factor, *S*(**G**_1_)/*S*(0), exhibits decaying oscillation and is characterized as a sharp peak at the shortest reciprocal-lattice vector. This sharp peak indicates that the water forms finite degree of ordering but the water molecules are not crystallized yet^17,23^. The degree of ordering is referred that to what extent the water molecules arrange like a solid^16,17,23,30,31^. And the degree of ordering within the water molecular near the walls can be measured by the value of the main peak of the in-plane static structure factor.

The larger of the value of the main peak of the in-plane static structure factor, the higher degree of ordering within the water molecular near the walls. The solid wall is a face-centered-cubic structure indicating that the solid atoms are arranged as a certain periodical manner, which generates a similar periodical potential field above the solid walls^41^. It has been showed that the ordering within the water molecular near the walls is induced by the periodical potential generated by the walls^16,23,30,31^. Larger water-solid interaction energy will induce more corrugated periodical potential, and the mobility of water molecules are more constrained by the periodical potential. In other words, the diffusion of water molecules becomes weaker leading to higher ordering, i.e., larger main peak of the in-plane static structure factor. Therefore, the slip length is less at stronger water-solid interaction energy. As the water-solid interaction energy decreases to *ε*_*wo*_ = 0.12424 Kcal/mol and 0.06212 Kcal/mol, the periodical potential induced by the walls becomes more smoother leading to weaker constraint of wall potential to the water molecule, i.e., the value of the main peak of the in-plane static structure factor becomes smaller, hence, larger slip.

**Figure 6:**
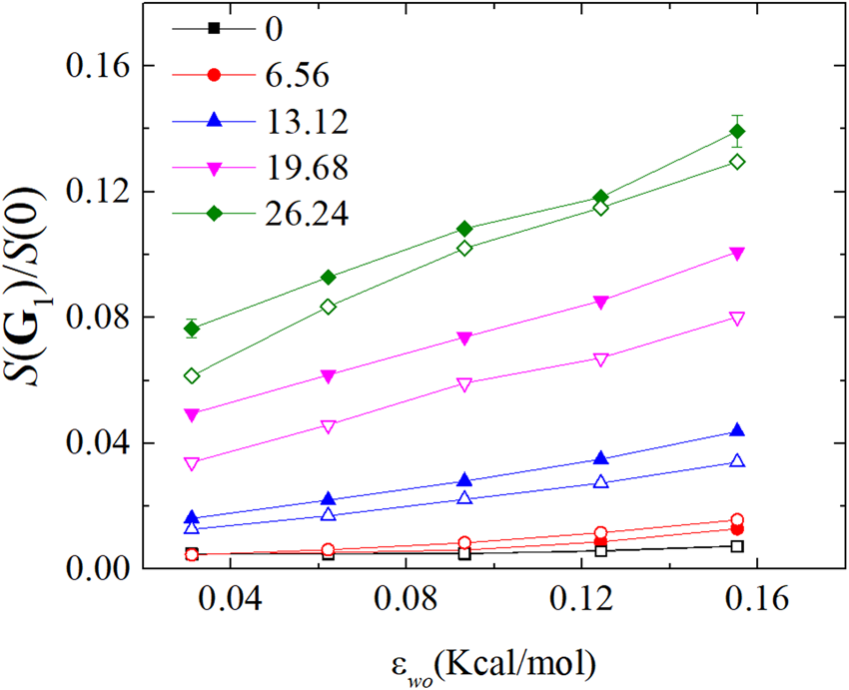
The normalized main peak of the in-plane static structure, *S*(**G**_1_)/*S*(0), at different water-solid interaction energy for indicated surface charge density. The closed and open data point indicate the slip length calculated at the positive and negative charge walls, respectively.

In Fig. 6, we present the normalized main peak of the in-plane static structure at different water-solid interaction energies for indicated surface charge density. It can be seen from Fig. 6 that the value of the normalized main peak of the in-plane static structure decreases with the decreasing water-solid interaction energy for given surface charge density. This is consistent with the slip behaviors presented in the Fig. 4. For given water-solid interaction energy, the presence of surface charge increases the normalized main peak of the in-plane static structure leading to higher degree of ordering structure within the water near walls. Therefore, the slip length decreases when the surface is charged and larger surface charge density produce less slip.

It can be seen from Fig. 6 that the dependence of the normalized main peak of the in-plane static structure on the water-solid interaction energy changes from nonlinear relationship to linear relationship, which induces the similar change of the dependence of slip length on the water-solid interaction energy. At the surface charge density, *CD* = 6.56 *μ*C/cm^2^, the positive charge produces smaller the normalized main peak of the in-plane static structure, (hence, larger slip length) than that by the negative charge. While, the negative charge produces smaller the normalized main peak of the in-plane static structure, (hence, larger slip length) than that by the positive charge for the surface charge density *CD* > 6.56 *μ*C/cm^2^. The detailed correlation between the slip length and the main peak of the in-plane static structure is presented below.

**Figure 7:**
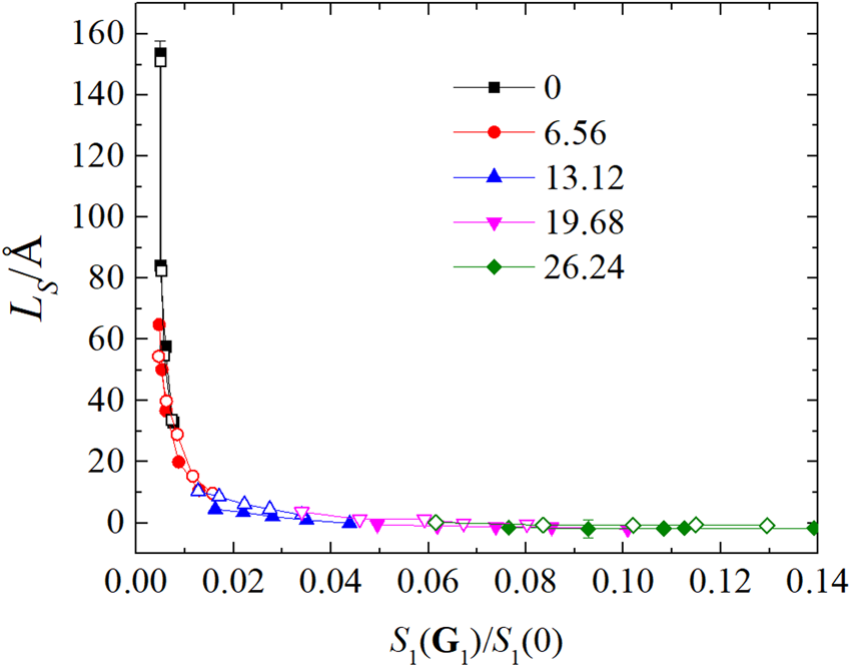
Behavior of the slip length, *L*_*S*_, as a function of the normalized main peak of the in-plane structure factor, *S*(**G**_1_)/*S*(0). The closed and open data point indicate the slip length calculated at the positive and negative charge walls, respectively.

The correlation between the value of the liquid water structure factor evaluated at the first reciprocal lattice vector **G**_1_ and the slip length is presented in Fig. 7. It can be seen from the Fig. 7 that the data of slip length as a function of the normalized main peak of the in-plane structure factor collapse onto a single curve for different water-solid interactions and surface charge densities. This scaling relationship between the slip length and the normalized main peak of the in-plane structure factor well holds in both cases of positive and negative charge surface. The similar scaling relationship was also found for simple fluid over atomistic smooth walls^17^. We extend this relationship to the situation of liquid water flowing past the charged surface.

## Conclusion

In this paper, the effect of surface charge density and water-solid interaction on the slip length and structure in a flow of liquid water was studied by non-equilibrium molecular dynamics simulations. A constant acceleration force was used to generate the Poiseuille-like flow condition. The upper and lower walls are positive and negative charged, respectively.

It was shown that the slip length decreases with the increasing of the surface charge density and water-solid interaction energy. And the ordering structure of liquid water near walls becomes higher with the increasing of the surface charge density and water-solid interaction. Interestingly, the positive charged surface induces less ordering structure and larger slip at the small surface charge density than that by the negative charged surface. While, at large surface charge density, the opposite correlation is observed. The presence of the surface charge increases the ordering structure of liquid water leading to the decrease of slip length. The increase of the surface charge density changes the dependence of the normalized main peak of the static structure factor on the water-solid interaction energy from nonlinear relationship to linear relationship leading to the similar change of the dependence of the slip length on the water-solid interaction. Furthermore, we find that the relationship between the slip length and the normalized main peak of static structure factor collapses onto a single curve for different water-solid interaction energy and surface charge density. The results of the present work open perspectives for modeling complex systems with combined effects of surface charge and wettability.
